# Nicorandil-Induced Hyperkalemia in a Uremic Patient

**DOI:** 10.1155/2012/812178

**Published:** 2012-10-18

**Authors:** Hung-Hao Lee, Po-Chao Hsu, Tsung-Hsien Lin, Wen-Ter Lai, Sheng-Hsiung Sheu

**Affiliations:** ^1^Division of Cardiology, Department of Internal Medicine, Kaohsiung Medical University Hospital, No. 100, Tzyou 1st Road, Kaohsiung 80756, Taiwan; ^2^Department of Internal Medicine, Faculty of Medicine, College of Medicine, Kaohsiung Medical University, No. 100, Shih-Chuan 1st Road, Kaohsiung 80708, Taiwan

## Abstract

Nicorandil is an antianginal agent with nitrate-like and ATP-sensitive potassium channel activator properties. After activation of potassium channels, potassium ions are expelled out of the cells, which lead to membrane hyperpolarization, closure of voltage-gated calcium channels, and finally vasodilation. We present a uremic case suffering from repeated junctional bradycardia, especially before hemodialysis. After detailed evaluation, nicorandil was suspected to be the cause of hyperkalemia which induced bradycardia. This case reminds us that physicians should be aware of this potential complication in patients receiving ATP-sensitive potassium channel activator.

## 1. Introduction

Nicorandil is an antianginal agent with nitrate-like and ATP-sensitive potassium (K_ATP_) channel activator properties. After activation of K_ATP_ channels, they expel potassium out of the cells, which lead to membrane hyperpolarization, closure of voltage-gated calcium channels, and finally vasodilation [1]. However, excessive activation of K_ATP_ channels may logically cause overt potassium efflux, which results in hyperkalemia. To our best knowledge, rare hyperkalemic cases due to K_ATP_ channels activator use were reported in the literatures' review. Mervyn Singer et al. had previously reported three cases of life-threatening hyperkalemia and hemodynamic disturbance due to K_ATP_ channels activation [2]. We present a uremic patient who developed hyperkalemia and junctional bradycardia after taking the nicorandil.

## 2. Case Presentation

A 51-year-old man with a past medical history of hypertension and end-stage renal disease (ESRD) requiring regular hemodialysis (three times per week) presented to the cardiovascular clinic due to progressive dyspnea and chest tightness for 2-3 months. During the first hospitalization, coronary angiography showed right coronary artery stenosis, but the patient hesitated about further intervention. After discharge, we prescribed nicorandil (5 mg three times per day) and aspirin (100 mg per day) at return visits. However, he still felt chest discomfort especially before his regular hemodialysis.

**Figure 1:**
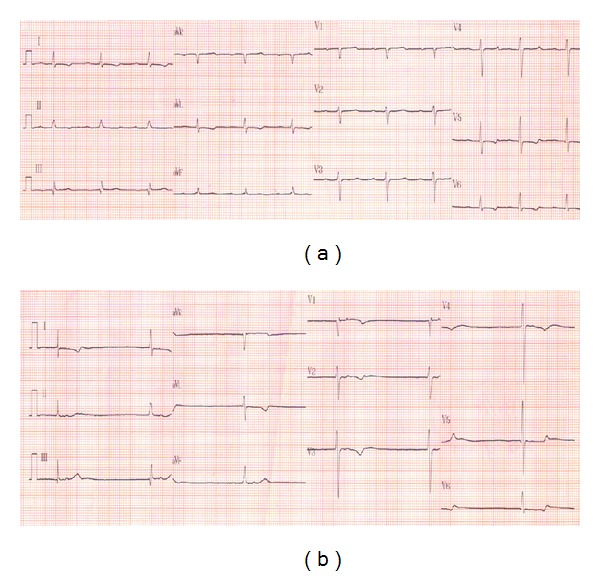
(a) Electrocardiography on admission showed first-degree atrioventricular block. (b) One day later when hyperkalemia was found, electrocardiography showed junctional bradycardia.

One month after the first angiography, he was admitted for percutaneous coronary intervention. However, chest tightness and dizziness were found in the next day after admission. Meanwhile, we found bradycardia (the heart rate between 30 and 40 beats per minutes) and relative hypotension (the blood pressure dropped from 188/79 mmHg to 102/79 mmHg). Electrocardiography (ECG) showed junctional bradycardia (Figure 1). Laboratory data revealed hyperkalemia (7.0 mmoL/L). Otherwise, we did not find significant abnormalities in other serum electrolytes (sodium 132 mmoL/L, ionic calcium 5.2 mmoL/L, and magnesium 2.7 mmoL/L), cardiac enzymes (CPK 305 IU/L, CK-MB 5.9 ng/mL, Troponin-I 0.14 ng/mL), serum glucose (90 mg/dL), and blood gas (pH 7.39). Under the tentative diagnosis of hyperkalemia-related bradyarrhythmia, we arranged emergent hemodialysis. Over the next few days, episodes of hyperkalemia recurred again and again. Although he had regular hemodialysis, the serum potassium elevated soon after each hemodialysis session.

**Figure 2:**
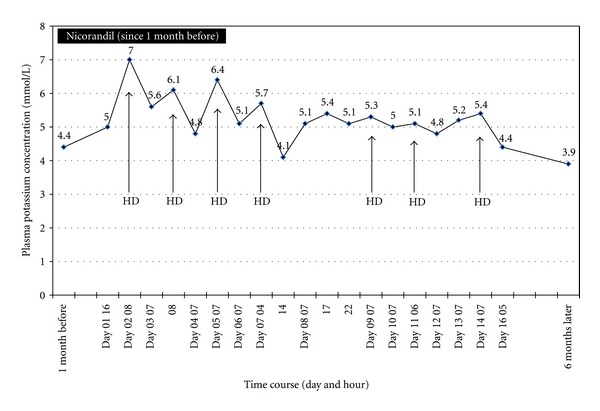
Recurrent hyperkalemia was found during nicorandil treatment. After cessation of nicorandil use, extreme hyperkalemia was not found again. HD (arrow): hemodialysis.

To exclude the possibility that his hemodialysis was not sufficiently removing potassium, serum potassium levels were examined before and after hemodialysis. We found serum potassium significantly decreased after each hemodialysis session. We also instructed him to avoid ingestion of high-potassium food such as starfruit, bananas, orange, raisins, and many vegetables (spinach and sweet potatoes). Even with above management, the hyperkalemia was still noted before next hemodialysis (Figure 2). In review of his medications, his only took aspirin and nicorandil recently. After reviewing adverse effects of these drugs, potassium channel syndrome caused by nicorandil was suspected. After cessation of nicorandil use, the serum potassium level decreased and there were no more bradyarrhythmia events. Then he was discharged uneventfully. No recurrent episode was found at follow-up visits, and the potassium level was 3.9 mmoL/L at 6 months later.

## 3. Discussion

The K_ATP_ channels are composed of two components, an inwardly rectifying potassium channel (Kir) pore subunit and the regulatory sulfonylurea-receptor (SUR). The pore confers ATP inhibition. The sulfonylurea receptor is the primary target for sulfonylureas, potassium channel openers, and nucleoside diphosphates. The K_ATP_ channels could open in response to certain physical stress (hypoxia, hypercapnia, acidosis, ATP depletion, etc.) or by drugs with K_ATP_ channel opening effect. However, glibenclamide could close the K_ATP_ chanenels after binding to sulfonylurea receptors [2–5]. 

The K_ATP_ channels are present in cardiomyocytes, skeletal muscle cells, vascular smooth muscle cells, pancreatic beta-cells, neurons, and mitochondria. In vascular smooth muscle cells, the K_ATP_ channels play roles in control of blood flow. After activation, they cause intracellular potassium efflux, which in turn results in membrane hyperpolarization, closure of voltage-gated calcium channels, and, ultimately, vasodilation. Besides, they may also contribute to vasodilation by enhancing release of nitric oxide (NO) from endothelium. The vasodilation occurred predominantly in small arterioles of coronary, mesenteric, renal, and skeletal muscle beds, which match blood flow to tissue needs [2, 5]. K_ATP_ channel also plays a central role in ischemic preconditioning (IPC). So opening of the myocardial K_ATP_ channel may have cardioprotective function against various stress, including ischemia and hypoxia [4].

Nicorandil is a novel antianginal agent with dual mechanisms of K_ATP_ channel activator and nitrate-like effect, which causes both arterial and venous vasodilations. Based on the Impact of Nicorandil in Angina (IONA) study, use of nicorandil can significantly improve in cardiovascular outcome in patients with stable angina [6]. 

The safety of nicorandil was proved in various studies [7, 8]. Arnold et al. have evaluated hemodynamic dose response of nicorandil in 42 patients, and they reported three patients developed transient symptomatic hypotension and bradycardia [9]. In the Safety Profile of Nicorandil-Prescription-Event Monitoring (PEM) cohort study, only 6 possible cases of bradycardia were reported among 13,260 patients from December 1994 to October 1996 [10]. However, hyperkalemia was not mentioned in these previous studies. 

In 1997, Montgomery et al. first reported that a 78-year-old man taking nicorandil had elevated serum potassium level and decreased cardiac pacemaker activity. After intravenous dextrose and insulin, spontaneous ventricular activity recovered within 10 minutes and then followed by sinus rhythm [11]. 

Singer et al. reported three cases developing severe life-threatening complications, including hyperkalemia, bradycardia, and hypotension, after using various K_ATP_ channel activators including ciclosporin, isoflurane, and nicorandil [2]. There was a poor response to conventional treatments. However, administration of glibenclamide promptly reversed these abnormalities. The glibenclamide binds to sulfonylurea-receptor subunits which act as K_ATP_ channel inhibitor. Singer et al. described it as “potassium channel syndrome,” which is associated with drug-related, excessive K_ATP_ channel activation that responds to inhibition by glibenclamide. 

Because the K_ATP_ channel could be activated by physical stress and medications, critical illness might potentiate the channel-opening effect of drugs, leading to hyperkalemia. Besides, it is known that uremic patients were prone to ATP depletion [12]. With several predisposing factors (ischemia and uremia), the patient developed hyperkalemia after nicorandil use. 

In previous studies, the nicorandil was not eliminated by renal excretion and the pharmacokinetic parameters are not significantly affected by renal impairment, so dose adjustment is not required [13, 14]. We had excluded other possible etiologies of hyperkalemia such as acidosis, high potassium diet, and other medications. The hyperkalemia was managed initially by hemodialysis, but extreme elevation of serum potassium level was found soon. In our case, we did not try to administrate glibenclamide to our patient due to ESRD. After cessation of nicorandil, the serum potassium level did not elevate significantly even before hemodialysis. 

In conclusion, we first reported a uremic patient who developed hyperkalemia after taking nicorandil. After detailed evaluation, we have excluded other possible etiologies and nicorandil-induced potassium channel syndrome was diagnosed. In addition to the anti-ischemic benefit, physicians should be aware of this potential complication in patients receiving K_ATP_ channel activator, nicorandil.
